# Supplementary material for: Cataract induction in an arthropod reveals how lens crystallins contribute to the formation of biological glass
Source: PLoS One. 2025 Jun 11;20(6):e0325229. doi: 10.1371/journal.pone.0325229 (PMC12157205; doi:10.1371/journal.pone.0325229)
Supplement: S1 File — (DOCX) [file pone.0325229.s001.docx]

**Supplementary material**

**
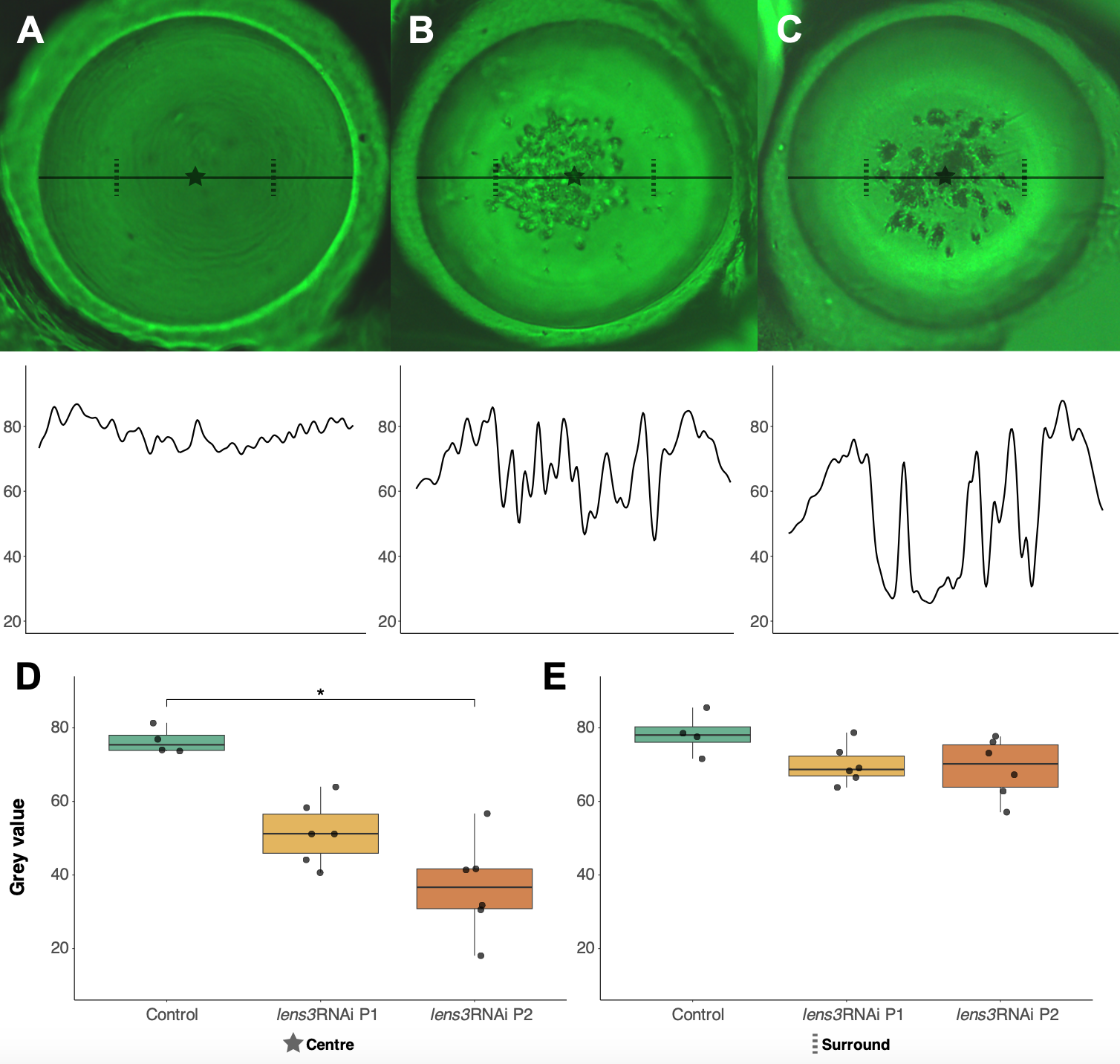
**

**Figure S1**. Lens back surface images and spatial quantification of cataracts. Top row depicts single diametric solid lines through the clear lens of a control (A) and cataractous lenses of *lens3*RNAi individuals (B; Probe1 and C; Probe 2). Middle row depicts corresponding densitometry traces based on pixel brightness values along all points on the selected lines, showing the location, extent and intensity of cataracts (method adapted from Seeberger et al., 2004 [[73]](https://paperpile.com/c/5CjFtz/9Cr9)). Extracted grey values from the centre (D; star) and surround (E; mean of dotted lines) of selected lines (*n_control_* = 4, *n_lens3RNAi P1_* = 6, *n_lens3RNAi P2_* = 6) are plotted in the bottom row. Pixel brightness values for control lenses were significantly higher than those with cataracts in the centre (ANOVA: *F*_2,13_ = 19.4, *p* < 0.001) but not the surround (ANOVA: *F*_2,13_ = 2.708, *p* + 0.1), illustrating prevalence of opacities towards lens centres.

**
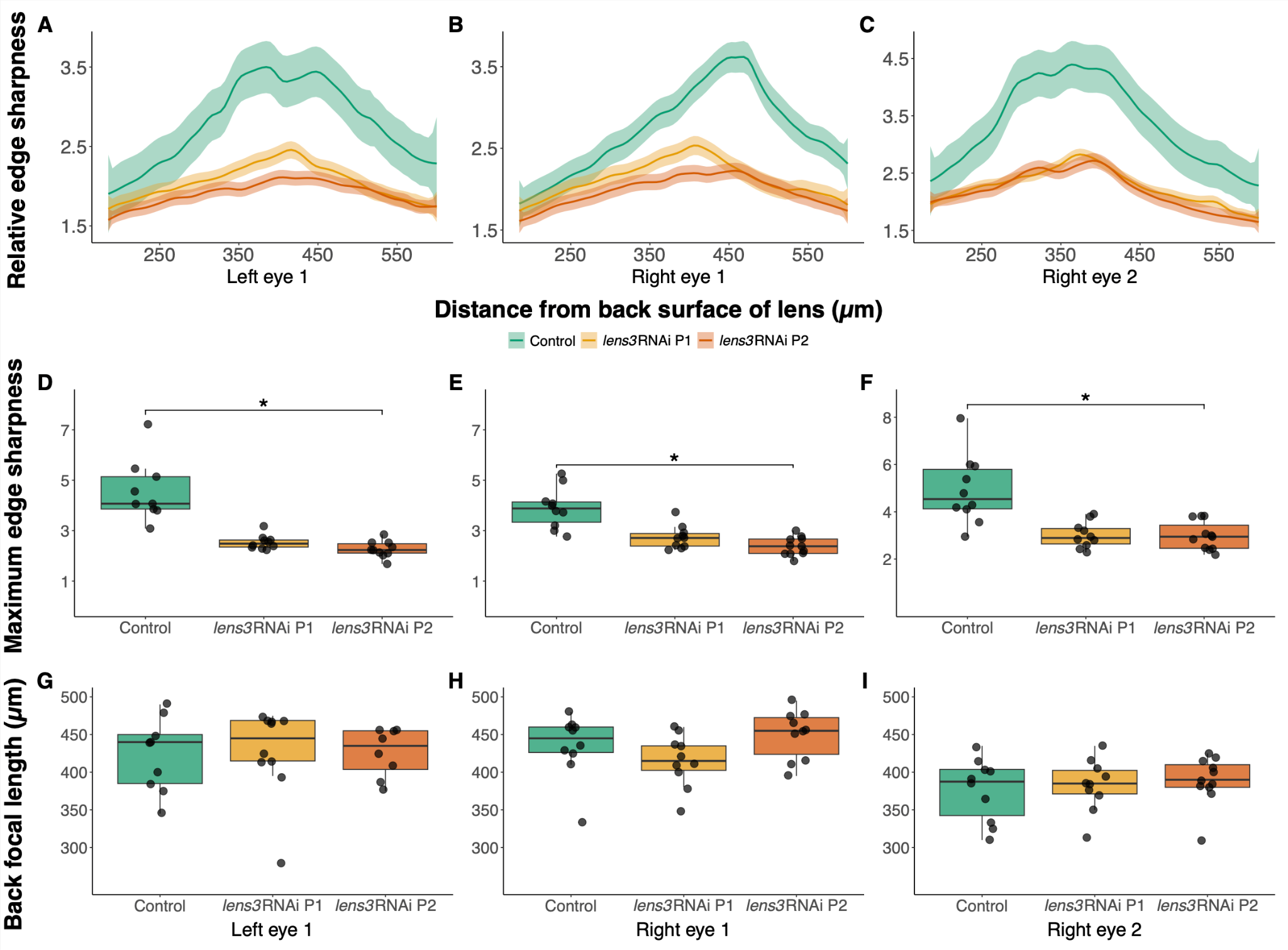
**

**Figure S2**. Assessment of lens optics through measurements of relative edge sharpness for left E1, right E1, and right E2. (A-C) Edge sharpness (LOESS) curves (*n_control_* = 9, *n_lens3RNAi P1_* = 10, *n_lens3RNAi P2_* = 11) show that the lenses of test individuals project blurry images compared to controls (shaded areas represent standard error). This is highlighted by a significant reduction in maximum edge sharpness (peak values extracted from individual curves) in lens3RNAi treated larvae (D-F; *n_control_* = 9, *n_lens3RNAi P1_* = 10, *n_lens3RNAi P2_* = 11; Kruskal-Wallis: d.f. = 2, 𝜒^2^_LE1_ = 19.7, *p*_LE1_ < 0.01; 𝜒^2^_RE1_ = 17.8, *p*_RE1_ < 0.01; 𝜒^2^_RE2_ = 14.8, *p*_RE2_ < 0.01;). (G-I) In contrast the back focal length, or the distance from the lens at which images are best focused (i.e., having highest edge sharpness), is not significantly different across groups (G-I; *n_control_* = 9, *n_lens3RNAi P1_* = 8, *n_lens3RNAi P2_* = 9; ANOVA: *F*_LE1; 2,25_ = 0.13, *p*_LE1_ = 0.87; *F*_RE1; 2,27_ = 2.20, *p*_RE1_ = 0.13; *F*_RE2; 2,28_ = 0.32, *p*_RE2_ = 0.72), suggesting that *lens3*RNAi does not cause detectable shifts in focal length.


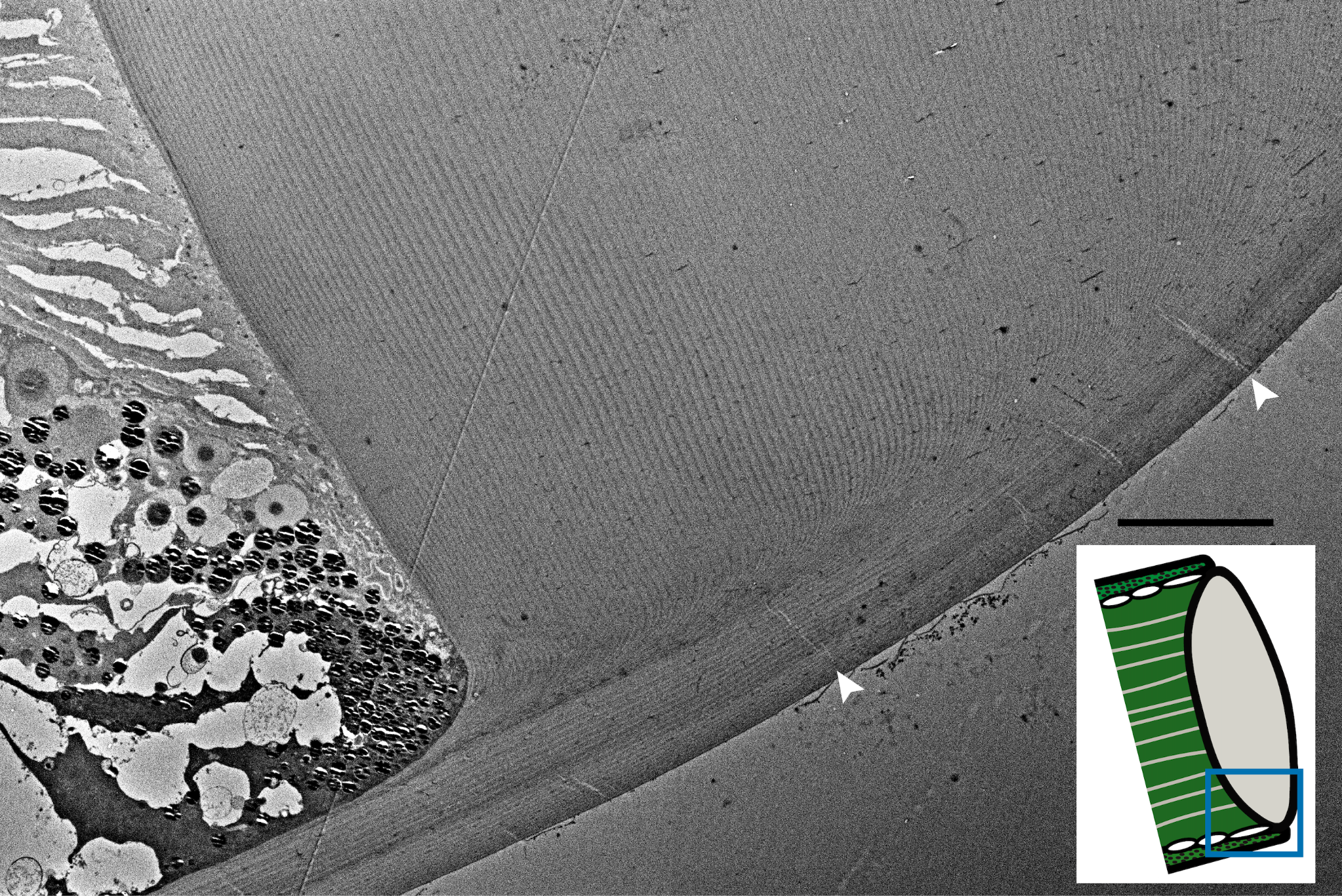


**Figure S3.** Opacities caused by reduced Lens3 levels are localised. In contrast to the proximal lens centre (Figure 5), the periphery and outer surface of *lens3*RNAi individuals show no defects, as exemplified in this TEM micrograph. Arrowheads point to pore canals typically found in arthropod lenses and cuticle [[1,2]](https://paperpile.com/c/5CjFtz/meBt+eEAj). Inset depicts the principal eye region that is visualised. Scale bar = 10µm.


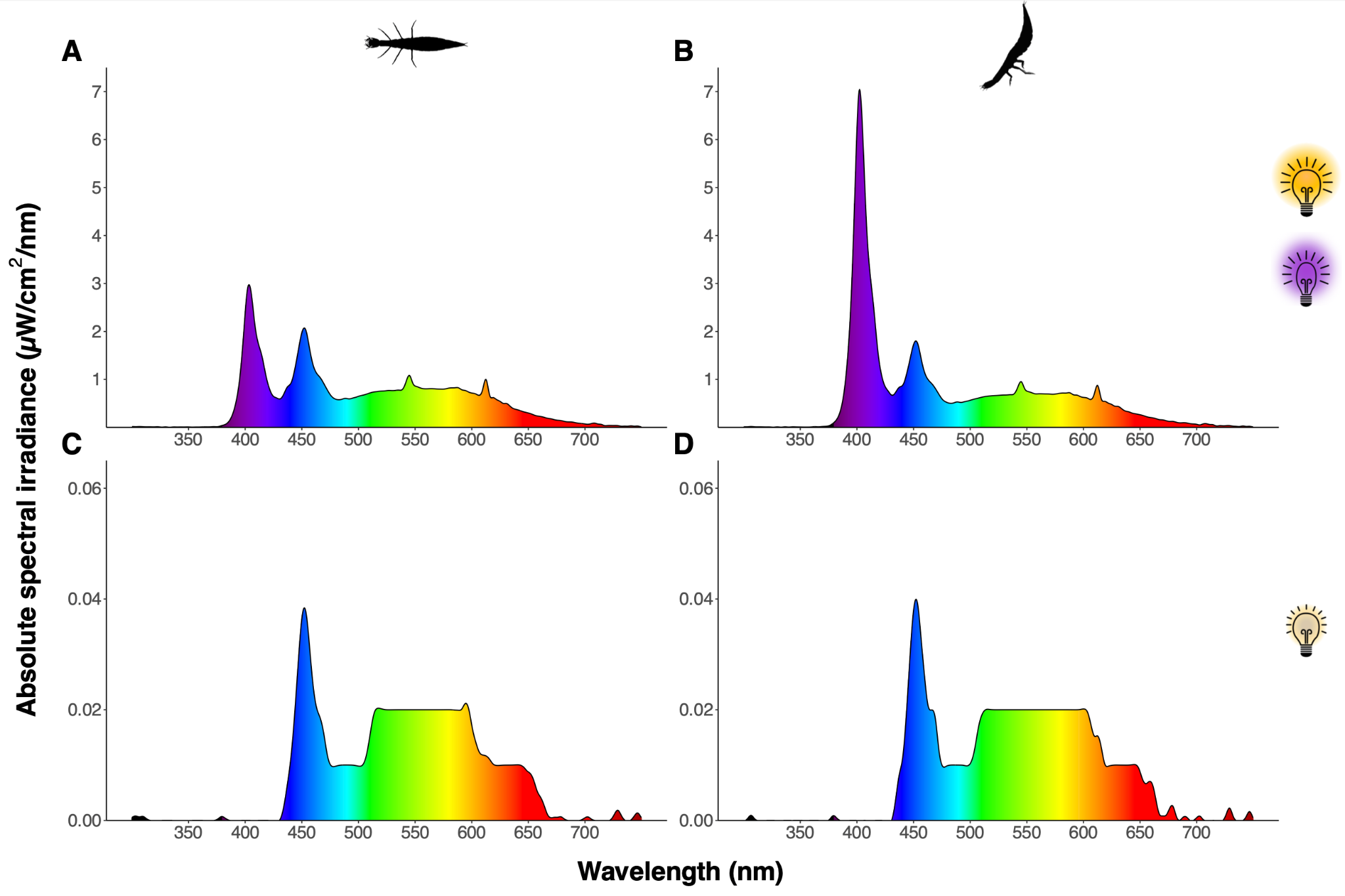


**Figure S4**. Spectral composition of lighting that was used in behavioural trials for assessment of larval hunting. Top row depicts horizontal (A) and vertical (B) arenas in bright white light supplemented with UV (horizontal arena = 6.27 x 10^14^ photons/cm^2^/s; vertical arena = 7.03 x 10^14^ photons/cm^2^/s). Bottom row depicts horizontal (C) and vertical (D) arenas in dim white light in the absence of UV supplementation (horizontal arena = 9.29 x 10^12^ photons/cm^2^/s; vertical arena = 9.29 x 10^12^ photons/cm^2^/s).


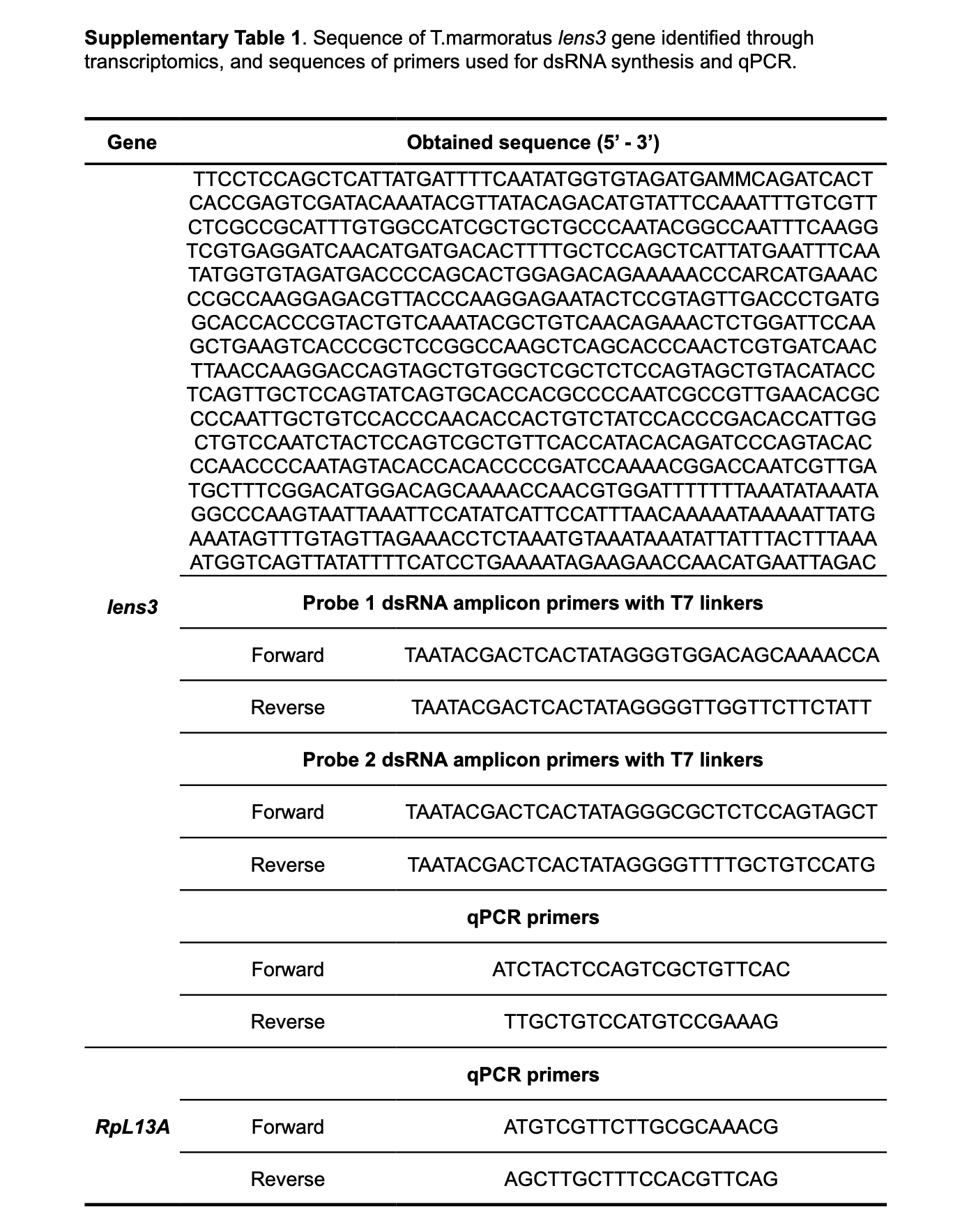


1. [Alagboso FI, Reisecker C, Hild S, Ziegler A. Ultrastructure and mineral composition of the cornea cuticle in the compound eyes of a supralittoral and a marine isopod. J Struct Biol. 2014;187: 158–173.](http://paperpile.com/b/5CjFtz/meBt)

2. [Meyer-Rochow VB, Yamahama Y. An anatomical and ultrastructural study of the eye of the luminescent millipede *Paraspirobolus lucifugus* (Gervais 1836) (Diplopoda, Spirobolida, Spiroboleliidae). Arthropod Struct Dev. 2022;69: 101171.](http://paperpile.com/b/5CjFtz/eEAj)
