# Supplementary material for: Cataract induction in an arthropod reveals how lens crystallins contribute to the formation of biological glass
Source: PLoS One. 2025 Jun 11;20(6):e0325229. doi: 10.1371/journal.pone.0325229 (PMC12157205; doi:10.1371/journal.pone.0325229)
Supplement: Attachment — Submitted filename: Dear Editor_PONE-D-25-03003_reviewer.pdf [file pone.0325229.s002.pdf]

Dear Editor,

This study by Mitra et al. explores the role of the Lens3 gene in lens development in *Thermonectus marmoratus* larvae, an arthropod species, using RNA interference (RNAi) to silence Lens3 and assess its effects on lens transparency, refractive properties, and visual function. The authors report that Lens3 knockdown induces cataract-like opacities, degrades image quality, and impairs hunting behavior under low-light conditions, while the eye's refractive state remains unchanged. This work is innovative and contributes valuable insights into lens biology and arthropod vision, though some issues require attention to enhance its scientific rigor and clarity.

Major Concerns:

- The manuscript speculates that Lens3 knockdown disrupts lens transparency via protein aggregates and chitin disorganization but lacks direct evidence or a clear mechanism. To clarify the mechanism, it would be beneficial to provide supporting data (e.g., biochemical assays) or explicitly note the current lack of evidence as a limitation for future research.
- The term "cataract" is used for lens opacities without sufficient quantitative or comparative justification. Strengthening the classification of lens opacities as "cataracts" by quantifying their size, density, and distribution, and comparing them to vertebrate cataracts, would enhance the analysis.
- Lens3 is not compared to lens proteins in other arthropods or vertebrates, limiting evolutionary insight. Adding a comparative analysis (e.g., sequence alignments or functional similarities) would help place Lens3 in a broader biological context.
- The unchanged refractive state despite image degradation is noted but underexplored. Expanding the discussion on why the refractive state remains unchanged despite image degradation, and exploring its implications for refractive development in arthropods versus vertebrates, would be beneficial.

Minor Concerns:

- The term "biological glass" is a key term in the title and text but is not defined, which may confuse readers. Adding a concise definition in the introduction would clarify its use in the context of lens biology.
- The use of "lens3RNAi" and "Lens3 knockdown" interchangeably may cause confusion. Using consistent terminology throughout the manuscript would improve clarity.

- In the cDNA preparation experiments, please clarify how you excluded the possibility of DNA contamination in the extracted RNA, as this could impact the accuracy of the qPCR results used to confirm Lens3 knockdown.
