# Supplementary material for: Cataract induction in an arthropod reveals how lens crystallins contribute to the formation of biological glass
Source: PLoS One. 2025 Jun 11;20(6):e0325229. doi: 10.1371/journal.pone.0325229 (PMC12157205; doi:10.1371/journal.pone.0325229)
Supplement: Attachment — Submitted filename: Response to review.docx [file pone.0325229.s003.docx]

Response to Review

Reviewer #1:
The article ‘Cataract induction in an arthropod reveals how lens crystallins contribute to the formation of “biological glass”’ (Amartya T. Mitra et al.) contains an interesting and original study of cataracts model in arthropods and can be recommended for publication with a few points.
Response: We thank the reviewer for their helpful comments and suggestions to improve our work. Specific points are addressed as follows.

The abstract is vaguely written and more data on the specific results obtained should be added.
Response: As suggested we have substantially expanded the abstract to include more details on the specific results obtained.

The abbreviation FD is superfluous, it occurs only 2 times and can be done without it.
Response: We have edited the text to remove this abbreviation.

There is a lack of information on the protein composition of arthropod lenses, are there other proteins besides Lens3, this should be discussed and literature data should be cited.

Response: Based on proteomics, we know that there are 10 main proteins that contribute to the lens, the majority of which are cuticular (Stahl et al., 2017b). There is also literature suggesting cuticular proteins tend to be a main contributor to insect lenses (Vannini et al., 2016; Stahl et al., 2017a; Chen et al., 2023) . As suggested, we have added this information and expanded this section (lines 113-125).

Also missing are comments on the structural features of the Lens3 protein and its properties, if these are known, they should be given.

Response: We have described Lens3 in lines 118-122 of the introduction, noting that it is a protein of the expansive RR-2 cuticular protein subfamily (Willis, 2010; Stahl et al., 2017) containing the highly conserved RR-2 chitin-binding domain. Unfortunately, further information on structural features of this protein are unknown. Using the known Lens3 amino-acid sequence, we attempted to model its structure using the deep learning model AlphaFold3, but outside of the conserved domain, this was predicted with high uncertainty, likely due to an incomplete sequence. Making further comments about structure would thus be beyond the scope of this manuscript.

A clear and concise conclusion is missing.

Response: We have updated the manuscript and added a conclusion section.

Reviewer #2:
This study by Mitra et al. explores the role of the Lens3 gene in lens development in Thermonectus marmoratus larvae, an arthropod species, using RNA interference (RNAi) to silence Lens3 and assess its effects on lens transparency, refractive properties, and visual function. The authors report that Lens3 knockdown induces cataract-like opacities, degrades image quality, and impairs hunting behavior under low-light conditions, while the eye's refractive state remains unchanged. This work is innovative and contributes valuable insights into lens biology and arthropod vision, though some issues require attention to enhance its scientific rigor and clarity.

Response: Thank you for your positive assessment of our science and the very helpful comments. We have carefully considered your feedback and have addressed specific comments below.

Major Concerns:
The manuscript speculates that Lens3 knockdown disrupts lens transparency via protein aggregates and chitin disorganization but lacks direct evidence or a clear mechanism. To clarify the mechanism, it would be beneficial to provide supporting data (e.g., biochemical assays) or explicitly note the current lack of evidence as a limitation for future research.

Response: We appreciate this comment and agree that further research is needed to confirm the nature of aggregates as protein. We have therefore modified the text to refer to these more generally as defects, and then make it clear in the discussion that we speculate (based on the ultrastructural evidence), that these relate to lens protein aggregates leading to chitin disorganisation.

The term "cataract" is used for lens opacities without sufficient quantitative or comparative justification. Strengthening the classification of lens opacities as "cataracts" by quantifying their size, density, and distribution, and comparing them to vertebrate cataracts, would enhance the analysis.

Response: We have updated the manuscript to strengthen our justification for referring to the lens opacities as cataracts, drawing comparisons with specific vertebrate cataract subtypes (see first paragraph of the results and Vasavada et al., 2004; Long et al., 2017; Fan et al., 2023). Additionally, for spatial quantification of the location, extent and distribution of opacities, we have added an additional supplementary figure (now Figure S1) with densitometry traces of lens images, a method adapted from Seeberger et al., 2004 for quantification of rodent lens opacities. Further comparisons with vertebrate cataracts are made in the discussion.

Lens3 is not compared to lens proteins in other arthropods or vertebrates, limiting evolutionary insight. Adding a comparative analysis (e.g., sequence alignments or functional similarities) would help place Lens3 in a broader biological context.

The following information has been added to the revised manuscript:

- A previous study revealed that Lens3 belongs to the RR-2 cuticular subfamily which also is the case for some of the *Drosophila melanogaster*, *Tribolium castaneum* and *Anopheles gambiae* lens proteins. (lines 118-122; Vannini et al., 2016; Stahl et al., 2017a; Chen et al., 2023)
- These proteins have been demonstrated to bind to chitin and are vastly different from the lens proteins of vertebrates which commonly are co-opted from stress protective proteins. (see first section of discussion)
- Based on proteomics, Lens3 was found to form heteromeric complexes with other *T. marmoratus* lens proteins (lines 122-125; Stahl et al., 2017b).

The unchanged refractive state despite image degradation is noted but underexplored. Expanding the discussion on why the refractive state remains unchanged despite image degradation, and exploring its implications for refractive development in arthropods versus vertebrates, would be beneficial.

Response: We thank the reviewer for asking us to clarify this. We explain the unchanged refractive state of the lens as what is expected with localized damage (rather than global damage) as the localized damage leaves large portions of the lens intact, allowing for normal refraction with the superposition of blurr that arises from light scatter in damaged areas. This information has been added to the discussion (lines 314-320).

Implications of form deprivation for refractive development of the eye in arthropods versus vertebrates are discussed in the last section of the discussion.

Minor Concerns:
The term "biological glass" is a key term in the title and text but is not defined, which may confuse readers. Adding a concise definition in the introduction would clarify its use in the context of lens biology.

Response: We have done as suggested and defined ‘biological glass’ in the introduction.
The use of "lens3RNAi" and "Lens3 knockdown" interchangeably may cause confusion. Using consistent terminology throughout the manuscript would improve clarity.

Response: As suggested, we have changed all instances in the text to “lens3RNAi”.

In the cDNA preparation experiments, please clarify how you excluded the possibility of DNA contamination in the extracted RNA, as this could impact the accuracy of the qPCR results used to confirm Lens3 knockdown.

Response: As we now outline in the methods, isolated RNA samples were treated with DNAse prior to cDNA preparation to exclude this possibility. To double check, we ran the RNA samples on a gel and found no evidence of DNA contamination. Lanes 1-3; controls (C12,13,15), lanes 4-6; *lens3*RNAi P1 (191F8, 191F9, 191F11); lane 7; ladder, lanes 8-9; *lens3*RNAi P2 (249F8, 249F9, 249F10)

Editorial comments

1) We reviewed the formatting guidelines and re-formatted the manuscript accordingly
2) As requested we removed the reference to our funding from the acknowledgement.
3) The statement: “The funders had no role in study design, data collection and analysis, decision to publish, or preparation of the manuscript." is correct.
4) We did not use LaTex
5) We added “Supplementary material” to the end of the manuscript to point towards the pdf that contains additional figures and a table
